# Reliability and validity of the ankle inversion discrimination apparatus during walking in individuals with chronic ankle instability

**DOI:** 10.3389/fphys.2023.1036194

**Published:** 2023-01-19

**Authors:** Xuerong Shao, Ming Kang, Lijiang Luan, Fawei Deng, Roger Adams, Tao Wu, Jia Han

**Affiliations:** ^1^ School of Exercise and Health, Shanghai University of Sport, Shanghai, China; ^2^ Department of Rehabilitation Medicine, Shanghai Fourth People’s Hospital, School of Medicine, Tongji University, Shanghai, China; ^3^ Department of Rehabilitation, Second Affiliated Hospital of Nanchang University, Nanchang, China; ^4^ Research Institute for Sports and Exercise, University of Canberra, Canberra, ACT, Australia; ^5^ College of Rehabilitation Sciences, Shanghai University of Medicine and Health Sciences, Shanghai, China; ^6^ Faculty of Health, Arts and Design, Swinburne University of Technology, Hawthorn, VIC, Australia

**Keywords:** chronic ankle instability, sports injury, proprioception, reliability, validity

## Abstract

**Purpose:** 1) to explore the test-retest reliability of a new device for measuring ankle inversion proprioception during walking, i.e., the Ankle Inversion Discrimination Apparatus—Walking (AIDAW) in individuals with or without Chronic Ankle instability (CAI); 2) to assess its discriminant validity in differentiating individuals with or without CAI; 3) to investigate its convergent validity by examining its association with Cumberland Ankle Instability Tool (CAIT) and the Y Balance Test (YBT).

**Methods:** For test-retest reliability, 15 participants with CAI and 15 non-CAI healthy controls were recruited. Participants completed the AIDAW test twice with a 7-day interval. The area under the receiver operating curve (AUC) was obtained as the AIDAW score. The intraclass correlation coefficient (ICC) and MDC_90_ were calculated. For the validity study, another 20 individuals with CAI and 20 non-CAI healthy controls were involved. The AIDAW scores were analyzed by an independent samples *t*-test, and the optimal cutoff value of AIDAW scores to best distinguish individuals with CAI was calculated by Youden’s index. Spearman or Pearson correlation analysis was used to analyze the correlation between AIDAW proprioceptive scores and the CAIT and final YBT scores.

**Results:** For test-retest reliability, the ICC values for the CAI, non-CAI, and the whole group were 0.755, 0.757, and 0.761 respectively. The MDC_90_ of the CAI and non-CAI group was 0.04 and 0.05. Regarding discriminant validity, the AIDAW proprioceptive discrimination scores in the CAI group were significantly lower than those in the non-CAI group (*p* = 0.003); and the cutoff score for distinguishing CAI from the non-CAI participants was 0.759. For convergent validity, the AIDAW scores were significantly correlated with the functional balance YBT final scores (*p* = 0.001) and the CAIT scores (*p* = 0.009).

**Conclusion:** The AIDAW is a reliable and valid device for evaluating ankle inversion proprioception during walking in individuals with and without CAI. AIDAW can be used as a clinical assessment tool to discriminate CAI from non-CAI individuals and to monitor effects of rehabilitation. The AIDAW proprioceptive discrimination scores were significantly and positively correlated with YBT and CAIT scores.

## Introduction

The ankle-foot complex is the only part of the human body that contacts the ground in most daily and sports activities, with this causing a variety of ankle injuries (Han et al., 2015). Lateral ankle sprain (LAS) is the most common ankle injury, with 2-7 people per 1000 people injured every year (Vriend et al., 2009; Waterman et al., 2010). However, although only 50% of patients go on to seek medical help (Doherty et al., 2016), nearly 40% of LAS will proceed to chronic ankle instability (CAI) (Miklovic et al., 2018). Individuals with CAI usually present with various physical deficits (Gribble et al., 2013), such as reduced range of motion (Hoch and McKeon, 2011), decreased muscle strength (Khalaj et al., 2020), altered muscle activation (Lin et al., 2021), and diminished ankle proprioception (Han et al., 2021), which together are associated with giving way sensations and recurrent ankle sprains (Alghadir et al., 2020).

Proprioception is the ability to perceive and integrate the position and movement sense of the body parts to determine their status in space (Han et al., 2016), and it plays an essential role in neuromuscular control. A number of studies have investigated the relationship between ankle proprioception and CAI. A systematic review and meta-analysis found that ankle proprioceptive deficit in CAI was not evident in all forms of proprioceptive assessment (Xue et al., 2021), suggesting that ankle proprioceptive deficits in CAI may be task-specific, and should be assessed under a meaningful context that mimics real life situations.

Indeed, Han et al. (2016) have argued that users of a proprioception test must consider its ecological validity in order for the test to reflect the natural working state of the proprioception system during normal function when interacting with the environment. Following this principle, they developed the ankle inversion discrimination apparatus for landing (AIDAL) for assessing ankle inversion proprioception during landing, because individuals with CAI usually experience giving away and recurrently sprain their ankles during landing (Han et al., 2021). Their results showed for the first time that an ankle proprioceptive measure—the AIDAL score—was significantly correlated with the ankle instability symptoms, measured by Cumberland Ankle Instability Tool (CAIT), which had a sensitivity of 80.7% and a specificity of 84.9% with a score less than 24 points the cutoff for identifying CAI patients (Li et al., 2021), and thus the AIDAL can be used to differentiate between individuals with and without CAI (Han et al., 2021).

In addition to landing, CAI individuals may also have difficulties in walking on uneven ground, as reflected by ankle function and symptoms questionnaires (Hiller et al., 2006). Therefore, investigation of ankle proprioception during walking may provide useful information for understanding the proprioceptive mechanisms underlying the neuromuscular deficit associated with CAI. Although there has been a robotized ankle-foot orthosis developed for measuring ankle proprioception during walking by the threshold to detection of passive motion method (Fournier Belley et al., 2016), the ecological validity of the device may be affected because only one foot was equipped with the device, which would lead to unequal weight on the lower limbs during walking. Although the active movement extent discrimination assessment (AMEDA) method is considered to have good ecological validity (Han et al., 2016), to date there is no reliable device for measuring ankle inversion proprioception during walking based on the AMEDA method.

Self-reported instability and balance deficit are common conditions in CAI individuals, which are related to the severity of CAI (Rosen et al., 2016). The CAIT is a patient-reported measure and has been used widely because it can reflect the degree of ankle instability (McGee, 2020). Research has shown ankle proprioception at landing (Han et al., 2021) and going downstairs (in press) to be associated with CAIT scores. Dynamic balance can be measured by the Y Balance Test (YBT) (Hertel et al., 2006; Ko et al., 2020). Although current studies have shown that CAI individuals have worse self-reported ankle instability and dynamic balance than healthy non-CAI individuals, the relationships between performance on a test of ankle proprioception during walking and self-reported ankle instability and dynamic balance have not been reported.

Accordingly, we developed a novel device for measuring ankle inversion proprioception during walking, i.e., the Ankle Inversion Discrimination Apparatus—Walking (AIDAW). The aims of this study were: 1) to assess the test-retest reliability of the AIDAW in individuals with and without CAI; 2) to assess the discriminant validity of AIDAW for individuals with and without CAI; and 3) to assess the convergent validity of AIDAW, by examining the relationship between the AIDAW scores, ankle instability symptom CAIT scores, and functional balance YBT scores.

## Materials and methods

### Participants

Seventy participants volunteered to take part in this research. For both the reliability and validity studies, the G* Power software was used to determine the sample size. For the reliability study, the G* Power software was used to calculate the sample, with power = 0.7, significance level = 0.05, intraclass correlation assuming the null hypothesis = 0.5, intraclass correlation assuming the alternative hypothesis = 0.75. The software showed the minimum sample size was 29. Accordingly, we recruited 30 participants (15 CAI participants and 15 non-CAI participants). The sample size for the validity study was also calculated by G* Power software (effect size = 0.9, power = 0.85, significance level = 0.05). The minimal sample size was determined as 38. Accordingly, another 40 participants (20 CAI participants and 20 non-CAI participants) were recruited. The inclusion criteria for the CAI group were: 1) a history of at least one ankle sprain with noticeable swelling and pain 12 months ago; 2) unable to perform daily activities for at least 1 day after the acute ankle sprain; 3) at least two ankle instability episodes within the past 6 months; 4) a score of less than 24 on the Chinese version of Cumberland Ankle Instability Tool (CAIT) (Li et al., 2021). The exclusion criteria were: 1) a history of lower extremity surgery; 2) a history of diseases affecting balance function. The demographic characteristics of participants are shown in Table 1. Their footedness was determined by the Chinese version of Waterloo footedness questionnaire (Revised) (Yang et al., 2018).

**TABLE 1:** Data from the study.

	Reliability study		Validity study					
	CAI	Non-CAI	CAI	Non-CAI	t	p	95%CI	Cohen’s d
Participants, n	15	15	20	20	—	—	—	—
Male: female, n	9:6	9:6	10:10	10:10	—	—	—	—
Tested foot (right: center), n	14:1	15:0	15:5	20:0	—	—	—	—
Age, y (mean ± SD)	21.80 ± 3.41	22.60 ± 1.99	21.55 ± 2.82	22.45 ± 2.16	—	—	—	—
Weight, kg (mean ± SD)	60.48 ± 5.95	59.90 ± 10.25	61.59 ± 9.42	61.85 ± 9.47	—	—	—	—
Height, m (mean ± SD)	1.69 ± 0.07	1.67 ± 0.10	1.688 ± 0.07	1.67 ± 0.08	—	—	—	—
CAIT score, point (mean ± SD)	17.40 ± 2.72	28.40 ± 1.55	16.05 ± 3.83	28.55 ± 1.40	13.71	<0.001	10.62–14.38	4.34
YBT, % (mean ± SD)	—	—	92.31 ± 6.61	97.37 ± 7.51	2.261	0.03	0.53–9.59	0.73
AIDAW score, unit (mean ± SD)	—	—	0.75 ± 0.04	0.80 ± 0.05	3.15	0.003	0.02–0.07	1.01

CAI: the Chronic Ankle Instability group; non-CAI: the non-Chronic Ankle Instability healthy group; 95%CI: 95% confidence interval; YBT: the final YBT, scores; CAIT: Cumberland Ankle Instability Tool. For the validity study, the independent samples *t*-test was used for analyzing differences between groups.

This study was approved by the Committee for Ethics in Human Research at Shanghai University of Sport (approval number: 102772021RT120). All the participants provided signed informed consent before the data was collected.

### Instruments

**FIGURE 1:**
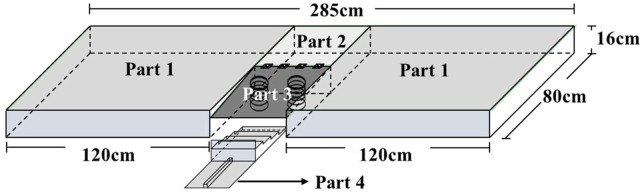
The design of the Ankle Inversion Discrimination Apparatus during walking (AIDAW) (Part 1: walking platforms; Part 2: bridging platform; Part 3: testing platform; Part 4: physical stops).

**FIGURE 2:**
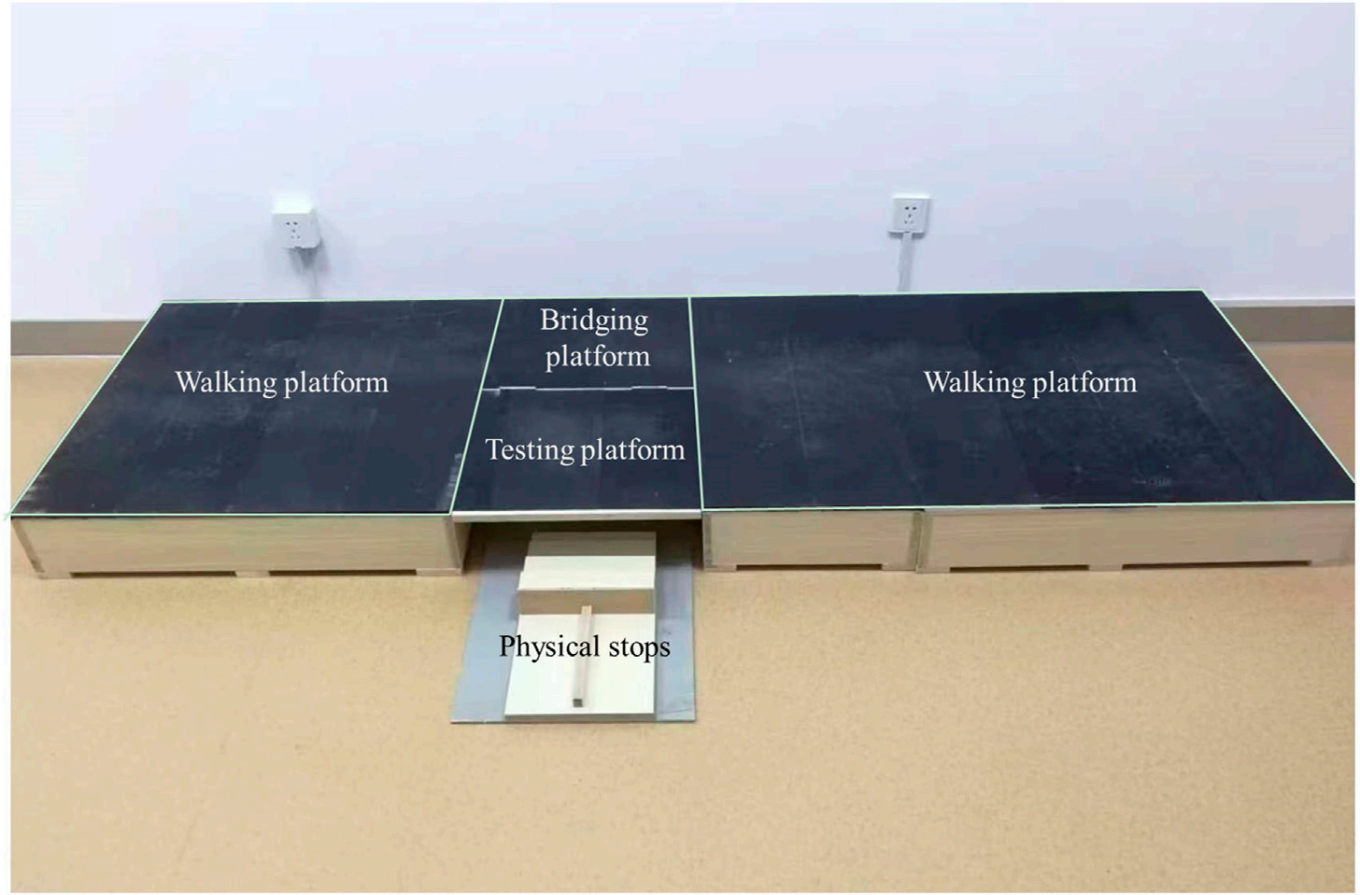
The genuine picture of AIDAW.

**FIGURE 3:**
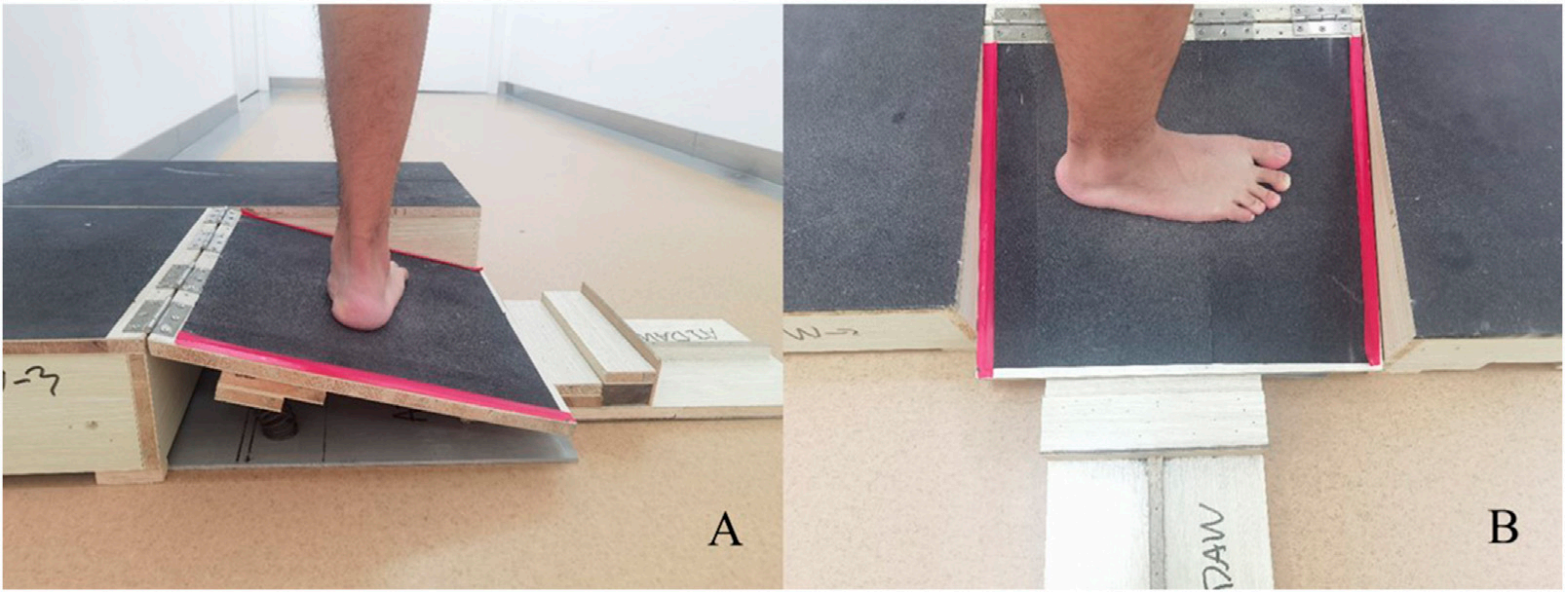
The testing platform in testing condition. **(A)** the left walking platform is removed in side view; **(B)** vertical view of testing platform.

**FIGURE 4:**
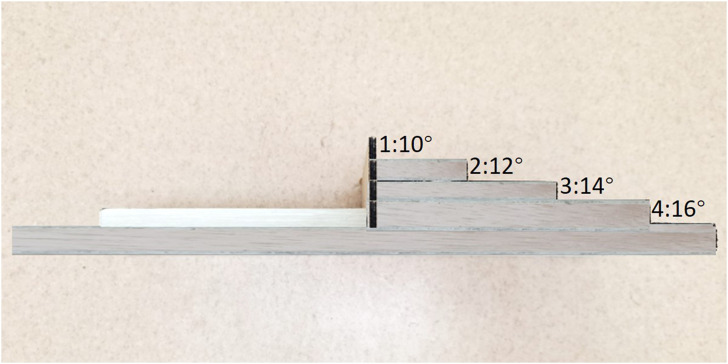
Lateral view of the physical stops that were slid in and out to generate the four possible ankle inversions of 10, 12, 14, and 16°.

A piece of new equipment, the Ankle inversion Discrimination Apparatus during Walking (AIDAW, Figure 1 and Figure 2), was purpose-built to assess ankle inversion movement discrimination sensitivity during walking. The AIDAW comprised four parts: part 1 was the walking platform, providing a normal walking surface before and after the test; part 2 was the bridging platform, connecting the walking platform and the testing platform; and part 3 was the testing platform (Figure 3), a horizontal wooden board held by two springs underneath, which could be tilted when participants stepped on it. The stiffness of the springs provided just sufficient support to hold the wooden board in horizontal when there was no additional weight on it, while allowing the testing platform to move freely to the predetermined tilt degree when participants walked across; and part 4 was the physical stops (Figure 4), which were used to adjust the tilt degree (10°, 12°, 14°, or 16°) of the testing platform in order to generate ankle inversions of 10° (number 1), 12° (number 2), 14° (number 3), or 16° (number 4) when participants stepped onto the testing platform. In everyday walking on flat surfaces, the maximum inversion angle is 8° (Smith et al., 2001), and thus the tilt degrees of the platform were greater than this but similar to those encountered when walking on uneven ground.

The Y Balance Test Kit™ was used to test the dynamic balance ability of the lower limbs (Plisky et al., 2009). The CAIT was used to measure patient self-reported ankle instability.

### Testing produces

For test-retest reliability study, participants performed two sessions of the AIDAW proprioception tests with an interval between them of 7 days. For the discriminant and convergent validity study, participants completed the AIDAW proprioception test and the YBT in random order. The testing foot for the CAI group was the one with lower CAIT score, or the dominant foot for the non-CAI group.

**FIGURE 5:**
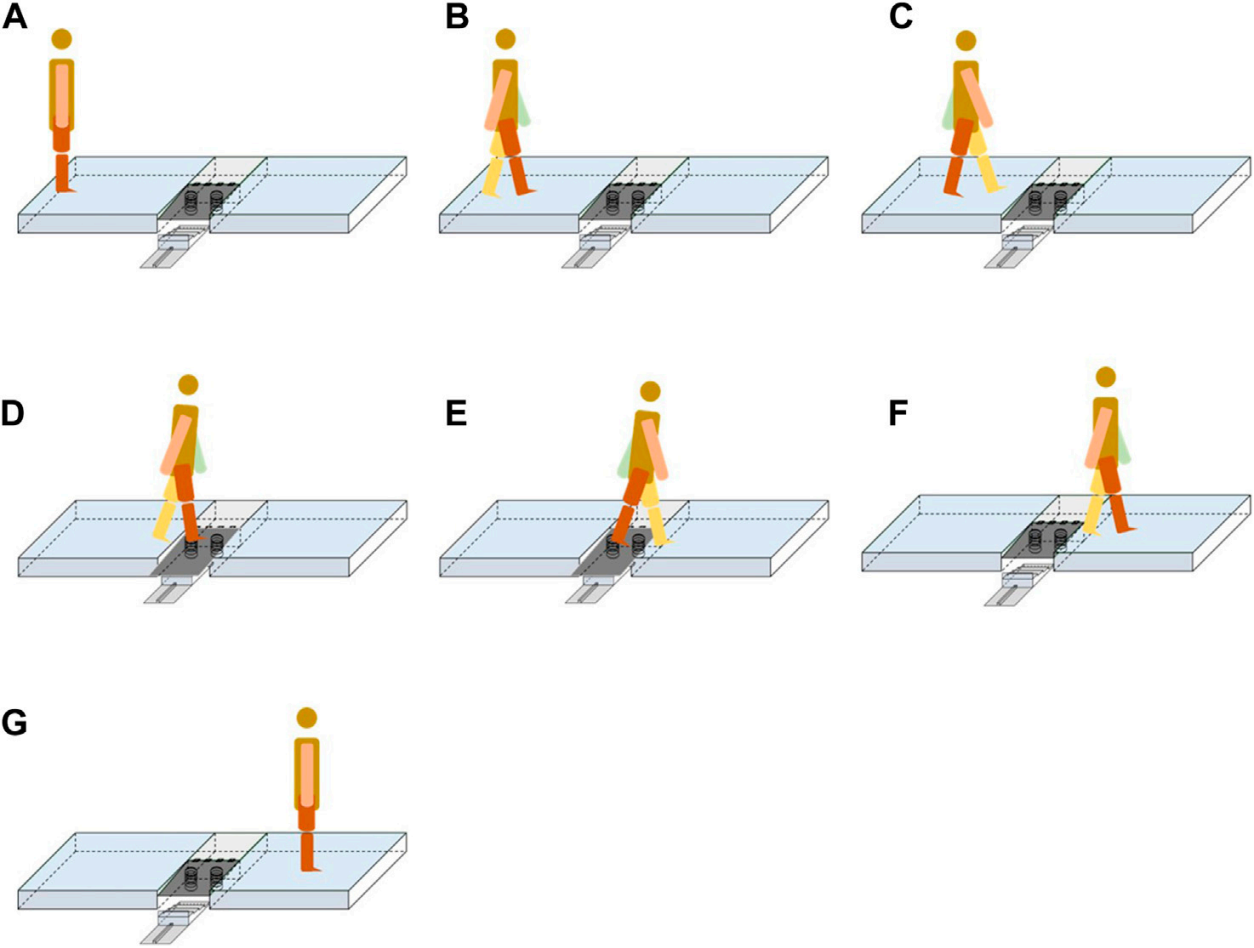
The test phase of the AIDAW. **(A)** starting position; **(B)** Step 1; **(C)** Step 2; **(D)** Step 3; **(E)** Step 4; **(F)** Step 5; **(G)** Step 6.

For the AIDAW test, participants were required to walk 6 steps at their normal, comfortable speed for each trial. Before testing, participants were allowed to adjust their starting point to ensure the testing foot could step onto the testing platform in step 3. During the test, participants were instructed to stand with bare feet on one side of the walking platform (depending on which foot was going to be tested), with eyes looking forward (Figure 5A). Participants started to walk when they heard the instruction “Go”, from initiating gait (Figure 5B; Step 1) to stepping across the testing platform (Figure 5C–F; Step 2-5), and continuing to walk for 6 steps in total. They then reported the number of the ankle inversion angle they perceived immediately after they completed the 6 steps (Figure 5G; Step 6). The walking direction was dependent on the tested side foot. When the tested foot was right side, the walking direction was from left to right ensuring the right foot could step onto the testing platform as in Figure 3, and *vice versa* such that when the tested foot was left side, the walking direction was from right to left.

Before data collection, there were 3 rounds of familiarization with the 4 ankle inversion positions, from 10 to 16° in order, and 12 trials in total, and participants were required to remember the feeling of each ankle inversion position. For the formal test, the 4 possible ankle inversion positions appeared randomly, and each inversion position appeared 10 times, for 40 trials in total. Participants were instructed to report their perceived ankle inversion position on each trial, without feedback on the correctness of their judgement. The actual presented positions and participants’ perceived positions were recorded on each trial for data analysis. Each participant would have 40 ankle inversion position stimuli and corresponding responses in total. The total test time was about 10 min.

**FIGURE 6:**
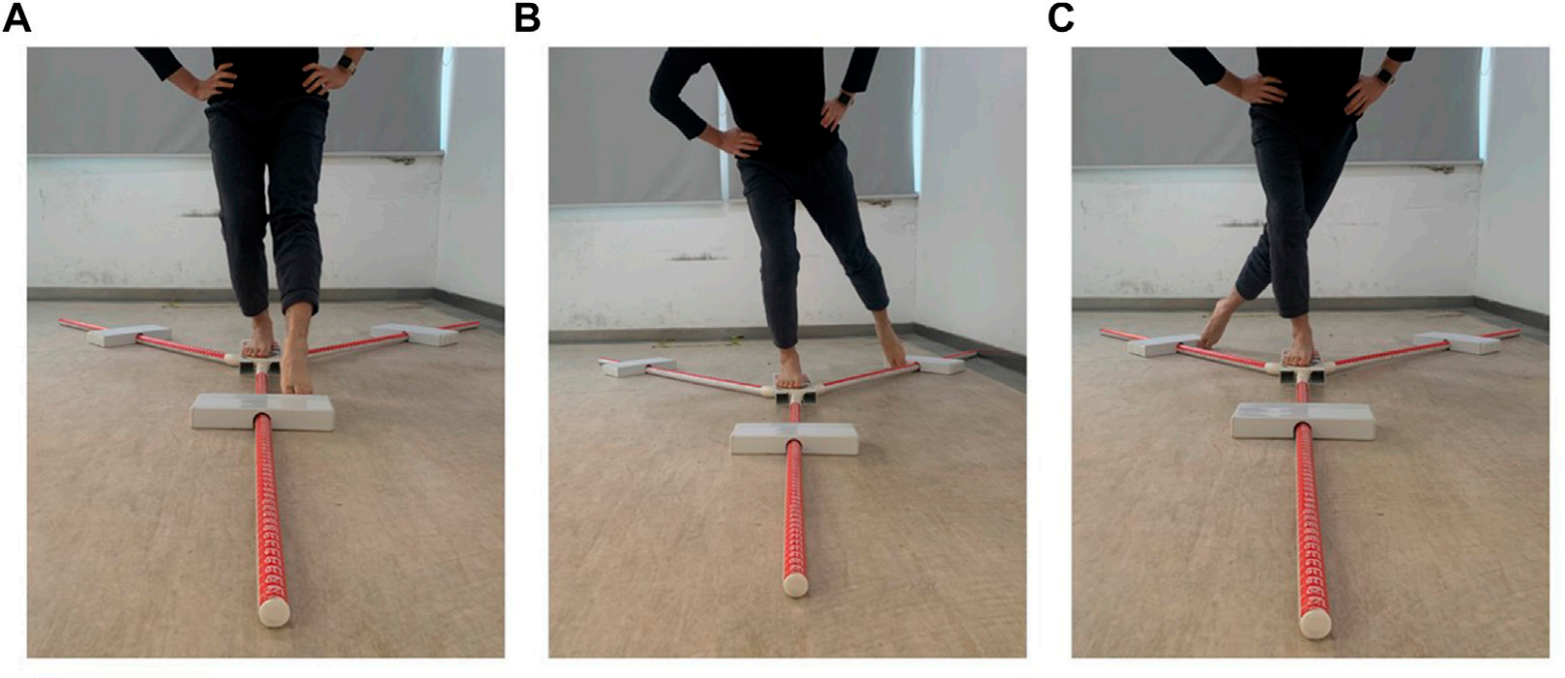
The Y balance test. **(A)** anterior direction; **(B)** posteromedial direction; **(C)** posterolateral direction.

For the YBT, participants stood on the standing block with the tested leg, and used the other foot to push the slide block as far as possible in anterior, posteromedial, and posterolateral directions (Figure 6) (Wilson et al., 2018). Before data collection, participants were given 2 rounds of familiarization. In the formal test, participants completed the test for 3 rounds, with 1 min rest in between. The distances achieved in 3 directions were recorded. The composite YBT scores were the sum of the distances (cm) in three directions, the normalized YBT scores were the composite YBT scores divided by three times the leg length (cm) multiplied by 100%. The leg length was measured from the anterior superior iliac spine to the most distal portion of the medial malleolus (Plisky et al., 2009). The final YBT scores were the average of the normalized YBT scores of three rounds of testing (Hudson et al., 2016).

The CAIT questionnaire was completed during demographic data collection, and the order of AIDAW and YBT tests was random.

### Statistical analysis

All data analyses were performed with SPSS version 24 (IBM Corporation Route100, Somers, NY10589). A *p*-value of <0.05 was set as statistical significance.

In order to calculate the AIDAW proprioceptive discrimination sensitivity score, the raw data of the 40 randomly assigned ankle inversion positions and their associated participant responses were entered into a 4 × 4 matrix representing the frequency with each response was made to each stimulus. Then the true positive rate (sensitivity) and the false positive rate (1-specificity) were calculated (Shao et al., 2022). The ROC curve is the plot of sensitivity against specificity. Non-parametric signal detection analysis was used to produce the Receiver Operating Characteristic (ROC) curves, and the mean value of the Area Under the Curve (AUC) values for each of the three pair-wise combinations (positions 1&2, 2&3, 3&4) was calculated as the AIDAW proprioceptive discrimination sensitivity score for each participant (Han et al., 2013). AUC values range from 0.5 to 1.0, with 0.5 representing chance level ability to discriminate between the 4 ankle inversion depths, and 1.0 meaning perfect discrimination sensitivity across the 4 ankle inversion depths.

For the test-retest study, the Intraclass Correlation Coefficient (ICC) and Bland-Altman plot were used. The ICC was calculated with a two-way fixed model, single measure type, and absolute agreement definition (Koo and Li, 2016). ICC values of ≤0.50, 0.50–0.75, 0.75–0.9 and >0.90, were taken as representing poor, moderate, good and excellent reliability, respectively (Koo and Li, 2016). The minimal detectable change (MDC_90_) was calculated with obtained ICC values using the following formula (Steffen and Seney, 2008; Hulzinga et al., 2020; Tao et al., 2021). The *SEM* employed was the standard error of the measurement, and *s* was the standard deviation of the measurements taken at the first time.

$$MDC_{90}=SEM\times \sqrt{2}\times 1.65$$

$$SEM=s\times \sqrt{1-ICC}$$

For assessing discriminant validity, an independent-groups *t*-test was used to test the difference in AIDAW scores between the CAI and the non-CAI groups. The cutoff score for discriminating between CAI and non-CAI individuals was calculated from the ROC curve, and the optimal cutoff score was calculated by the maximum value of Youden’s index (Tao et al., 2021), which is the maximum difference between sensitivity and 1-specificity.

For convergent validity, YBT and CAIT were analyzed. The difference analysis of YBT and CAIT between the CAI group and healthy non-CAI group was analyzed by independent samples *t*-test, the relationship between the AIDAW scores and the CAIT scores was tested by Spearman’s correlation analysis, and the relationship between the AIDAW scores and the final YBT scores was tested by Pearson’s correlation analysis. Correlation Coefficient values between 0.00–0.10, 0.10–0.39, 0.40–0.69, 0.70–0.89 and 0.90–1.00, were classified as negligible, weak, moderate, strong and very strong correlation, respectively (Schober et al., 2018).

## Results

**FIGURE 7:**
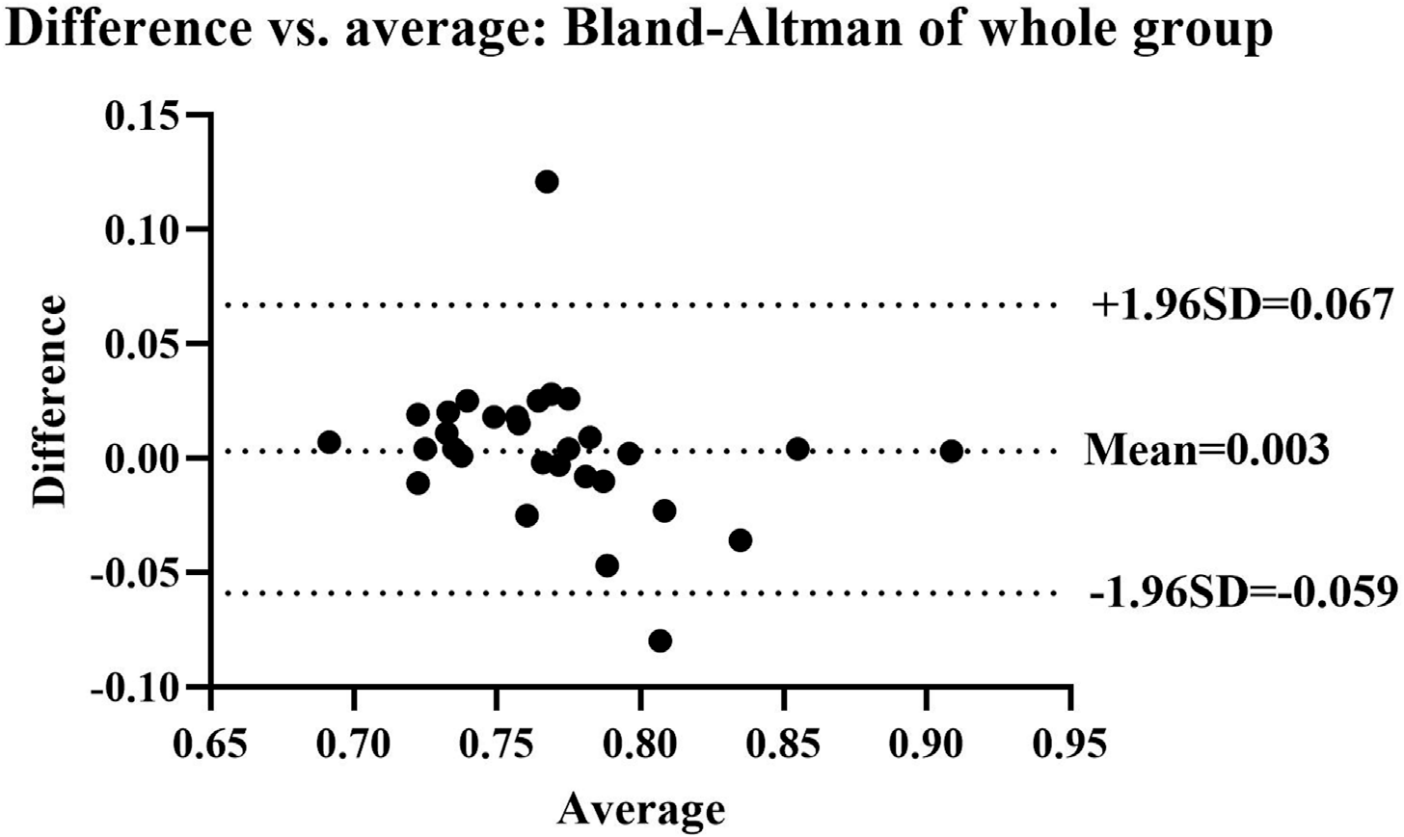
Bland-Altman plot showing agreement between the first and second AIDAW tests of the whole group. The mean difference score was 0.003, and the 95% limits of agreement were at −0.059 and 0.067. Two points fall beyond the 95% limits.

For test-retest reliability, ICC values of the CAI, non-CAI, and whole group were 0.755 (95%CI = 0.423–0.910), 0.757 (95%CI = 0.425–0.911), and 0.761 (95%CI = 0.557–0.879), respectively. Bland-Altman plots of the whole group are shown in Figure 7. The MDC_90_ values for the CAI and the non-CAI group were 0.04 and 0.05.

Regarding discriminant validity, the AIDAW scores in the CAI group were found to be significantly lower than in the non-CAI group (Table 1). The optimal cutoff score was 0.759 (sensitivity 60%, and specificity 85%) for distinguishing CAI from non-CAI participants.

For convergent validity, the difference analysis between the CAI group and healthy non-CAI group is shown in Table 1. Both the final YBT scores and CAIT scores of the CAI group were significantly worse than in the non-CAI group. Results of the correlation analysis showed that the AIDAW scores were moderately correlated with the functional balance YBT total scores (*r* = 0.51, *p* = 0.001) and the ankle instability symptoms CAIT score (*r*_*s*_ = 0.41, *p* = 0.009).

Although the YBT scores were significantly correlated with the AIDAW scores, the YBT was not ideal for discriminating CAI from non-CAI participants (AUC = 0.678, *p* = 0.055).

## Discussion

The test-retest reliability study showed that the ICC values for AIDAW were in the good reliability range between 0.70–0.90 in the CAI, non-CAI, and the whole group, and Bland-Altman plots showed good test-retest results in these groups. Previous research has reported comparable test-retest reliability for movement discrimination assessment devices. Specifically, the ICC values were 0.70–0.80 for CAI and non-CAI groups tested using the AIDAL (Han et al., 2021), and ICC values were 0.70–0.88 for the young and elderly groups when assessing their postural sway movement discrimination sensitivity using the Sway discrimination apparatus (SwayDA) (Chen et al., 2019). Therefore, this novel AIDAW method can be considered to be reliable for assessing ankle proprioception during walking in individuals with and without CAI.

The MDC value is the minimum change above the measurement error threshold needed for evidence of real effect (Haley and Fragala-Pinkham, 2006). Only when the testing value is changed above the MDC value can the change value be considered as the effect of treatment or intervention (Portney, 2020). In the current study, the AIDAW MDC_90_ values were similar to those of the AIDAL method (0.04 for both the CAI group and the non-CAI groups) (Han et al., 2021). These results suggest that the measurement error was small and that when the AIDAW is used as an evaluation tool for rehabilitation of ankle proprioception, an improvement of the AIDAW proprioceptive score greater than 0.04 can be considered as true change. To date, however, there has been no report regarding the MDC value of other proprioceptive measures for CAI, such as JPS and TTDPM.

Concerning the discriminant validity study, the AIDAW scores of the CAI group were significantly lower than for the non-CAI group. This result is consistent with what has been found in AIDAL testing (Han et al., 2021; Yu et al., 2021). The AIDAW cutoff score for discriminating CAI individuals and non-CAI individuals was 0.759, with sensitivity of 60% and specificity of 85%. To be specific, if a participant obtained an AUC score of AIDAW higher than 0.759, the participant had an 85% probability of being a non-CAI individual; in contrast, if the AUC score was lower than 0.759, then there was a 60% probability of them being an individual with CAI. Therefore, the AIDAW cutoff score could be used to facilitate decision-making by clinicians when identifying CAI individuals and in monitoring any effects of a physiotherapy program targeting ankle proprioceptive impairment associated with CAI.

For the convergent validity study, the correlation between the AIDAW score and CAIT score was moderate, also similar to that observed with the AIDAL (Han et al., 2021). In comparison, Marcos et al. (de Noronha et al., 2007) found the CAIT had a negligible or weak correlation with ankle proprioceptive acuity as measured by TTDPM. This discrepancy may be due to the difference in the ecological validity of the two types of the proprioceptive testing methods employed. Han et al. (2020) argued that for a person functioning in their environment there is passively imposed and actively obtained proprioception. The TTDPM method assesses imposed proprioception. During this test, participants are usually strapped to prevent other parts of the body from moving, and their vision and audition are blocked to eliminate other sensory input. Then the ankle joint is passively moved slowly by a motor, and participants are required to detect any movement imposed. In contrast, the AMEDA, AIDAL and the current AIDAW methods have been used for testing obtained proprioception, where the tests are conducted in functional positions, such as full-weight bearing, landing, and walking, with general visual and auditory information available for the brain to integrate and discriminate a series of ankle movement positions. The CAIT questionnaire is used to evaluate patient-perceived ankle instability during functional activities, such as walking, jumping and cutting. Therefore, the ecological validity of TTDPM method is relatively low and thus it is difficult to relate the scores of this test to human functional performance. Based on this fact, a recent systematic review and meta-analysis recommended that actively obtained proprioceptive methods, with higher ecological validity, be used for identifying proprioceptive impairments associated with CAI (Xue et al., 2021). In this case, the AIDAW method fulfills the ecological validity principle for developing obtained proprioception related to functional movements.

In addition, we also found moderate correlation between the AIDAW scores and the final YBT scores. YBT performance reflects dynamic balance ability, which is fundamental for exercise and sport performance. To maintain balance, the brain has to integrate proprioception, vision and vestibular information (Sturnieks et al., 2008). The foot-ankle complex is the only body part that contacts the ground, and ankle proprioception is arguably an important component in balance control (Han et al., 2015). The significant correlation between the AIDAW scores and YBT scores observed here supports this notion, and suggests that it is essential to assess ankle proprioceptive acuity using the AIDAW in CAI rehabilitation. In addition, future studies should explore the effects of physiotherapy programs on ankle proprioception during walking, and any possibly associated effects on dynamic balance control and patient-perceived symptoms, in individuals with CAI. The results from the YBT in our study are consistent with those reported in a previous study (Ko et al., 2020), where CAI patients showed significantly poorer YBT performance compared to non-CAI participants. In contrast, other research has found that CAI patients with a mechanically instable ankle performed equally well as those in the non-CAI control group (Wenning et al., 2021). In their study, participants were athletes wearing socks, who would have sport training experience and may have used the additional tactile input at the foot-ankle complex to compensate in YBT performance. Another possibility that might account for this inconsistency was that Wenning et al. (2021) used body height to normalize the YBT scores, while Ko et al. (2020) and the present study used lower limb length. Future studies may explore the effect of training experience and footwear on YBT in individuals with CAI, and compare the difference between data sets normalized using different methods.

## Strengths and limitations

The AIDAW is the first tool developed for measuring ankle proprioception during walking based on AMEDA methods which could be used to identify CAI in clinical practice. In this study, we established the MDC values for CAI and non-CAI individuals, values which could be used to confirm a true and clinically important change. This study also found that ankle proprioception was related to dynamic balance ability and perceived instability, suggesting its importance in lower limb control. Therefore, the results provide essential information for use of the AIDAW in clinical management of CAI. However, in the current study only young participants were recruited, so the results here may not be generalized to other populations, such as older participants.

In addition, compared to previous proprioceptive methods where participants were strapped on a machine and their vision and audition were blocked, the current AIDAW test was conducted under functional walking conditions, and thus the ecological validity of the test has been optimized. However, only six steps were performed, which is different from the number taken in normal walking activity.

## Conclusion

The novel AIDAW device is reliable and valid for evaluating ankle inversion proprioception during walking for individuals with and without CAI. Ankle inversion proprioception was significantly impaired in individuals with CAI, and the AIDAW can be used as a discrimination tool for separating CAI from non-CAI individuals. The AIDAW scores were significantly and positively associated with YBT and CAIT scores, suggesting that rehabilitation programs targeting ankle proprioception impairment may be beneficial in improving functional performance and symptoms in individuals with CAI.

## Data Availability

The original contributions presented in the study are included in the article/Supplementary Material, further inquiries can be directed to the corresponding authors.

## References

[B1] Alghadir A. H. Iqbal Z. A. Iqbal A. Ahmed H. Ramteke S. U. (2020). Effect of chronic ankle sprain on pain, range of motion, proprioception, and balance among athletes. Int. J. Environ. Res. Public Health 17 (15), 5318. 10.3390/ijerph17155318 32718066 PMC7432694

[B2] Chen Z. Han J. Waddington G. Adams R. Witchalls J. (2019). Somatosensory perception sensitivity in voluntary postural sway movements: Age, gender and sway effect magnitudes. Exp. Gerontol. 122, 53–59. 10.1016/j.exger.2019.04.013 31029824

[B3] de Noronha M. Refshauge K. M. Kilbreath S. L. Crosbie J. (2007). Loss of proprioception or motor control is not related to functional ankle instability: An observational study. Aust. J. Physiother. 53 (3), 193–198. 10.1016/s0004-9514(07)70027-2 17725477

[B4] Doherty C. Bleakley C. Hertel J. Caulfield B. Ryan J. Delahunt E. (2016). Recovery from a first-time lateral ankle sprain and the predictors of chronic ankle instability: A prospective cohort analysis. Am. J. Sports Med. 44 (4), 995–1003. 10.1177/0363546516628870 26912285

[B5] Fournier Belley A. Bouffard J. Brochu K. Mercier C. Roy J. S. Bouyer L. (2016). Development and reliability of a measure evaluating dynamic proprioception during walking with a robotized ankle-foot orthosis, and its relation to dynamic postural control. Gait Posture 49, 213–218. 10.1016/j.gaitpost.2016.07.013 27450673

[B6] Gribble P. A. Delahunt E. Bleakley C. Caulfield B. Docherty C. L. Fourchet F. (2013). Selection criteria for patients with chronic ankle instability in controlled research: A position statement of the international ankle consortium. J. Orthop. Sports Phys. Ther. 43 (8), 585–591. 10.2519/jospt.2013.0303 23902805

[B7] Haley S. M. Fragala-Pinkham M. A. (2006). Interpreting change scores of tests and measures used in physical therapy. Phys. Ther. 86 (5), 735–743. 10.1093/ptj/86.5.735 16649896

[B8] Han J. Adams R. Waddington G. (2020). Imposed" and "obtained" ankle proprioception across the life span-Commentary on Djajadikarta et al. J. Appl. Physiol. 129 (3), 533–534. 10.1152/japplphysiol.00541.2020 32886026

[B9] Han J. Anson J. Waddington G. Adams R. Liu Y. (2015). The role of ankle proprioception for balance control in relation to sports performance and injury. Biomed. Res. Int. 2015, 842804. 10.1155/2015/842804 26583139 PMC4637080

[B10] Han J. Waddington G. Adams R. Anson J. (2013). Ability to discriminate movements at multiple joints around the body: Global or site-specific. Percept. Mot. Ski. 116 (1), 59–68. 10.2466/24.10.23.PMS.116.1.59-68 23829134

[B11] Han J. Waddington G. Adams R. Anson J. Liu Y. (2016). Assessing proprioception: A critical review of methods. J. Sport Health Sci. 5 (1), 80–90. 10.1016/j.jshs.2014.10.004 30356896 PMC6191985

[B12] Han J. Yang Z. Adams R. Ganderton C. Witchalls J. Waddington G. (2021). Ankle inversion proprioception measured during landing in individuals with and without chronic ankle instability. J. Sci. Med. Sport 24 (7), 665–669. 10.1016/j.jsams.2021.02.004 33632662

[B13] Hertel J. Braham R. A. Hale S. A. Olmsted-Kramer L. C. (2006). Simplifying the star excursion balance test: Analyses of subjects with and without chronic ankle instability. J. Orthop. Sports Phys. Ther. 36 (3), 131–137. 10.2519/jospt.2006.36.3.131 16596889

[B14] Hiller C. E. Refshauge K. M. Bundy A. C. Herbert R. D. Kilbreath S. L. (2006). The Cumberland ankle instability tool: A report of validity and reliability testing. Arch. Phys. Med. Rehabil. 87 (9), 1235–1241. 10.1016/j.apmr.2006.05.022 16935061

[B15] Hoch M. C. McKeon P. O. (2011). Joint mobilization improves spatiotemporal postural control and range of motion in those with chronic ankle instability. J. Orthop. Res. 29 (3), 326–332. 10.1002/jor.21256 20886654

[B16] Hudson C. Garrison J. C. Pollard K. (2016). Y-balance normative data for female collegiate volleyball players. Phys. Ther. Sport 22, 61–65. 10.1016/j.ptsp.2016.05.009 27583650

[B17] Hulzinga F. Nieuwboer A. Dijkstra B. W. Mancini M. Strouwen C. Bloem B. R. (2020). The new freezing of gait questionnaire: Unsuitable as an outcome in clinical trials? Mov. Disord. Clin. Pract. 7 (2), 199–205. 10.1002/mdc3.12893 32071940 PMC7011794

[B18] Khalaj N. Vicenzino B. Heales L. J. Smith M. D. (2020). Is chronic ankle instability associated with impaired muscle strength? Ankle, knee and hip muscle strength in individuals with chronic ankle instability: A systematic review with meta-analysis. Br. J. Sports Med. 54 (14), 839–847. 10.1136/bjsports-2018-100070 31937576

[B19] Ko J. Wikstrom E. Li Y. Weber M. Brown C. N. (2020). Performance differences between the modified star excursion balance test and the Y-balance test in individuals with chronic ankle instability. J. Sport Rehabil. 29 (6), 748–753. 10.1123/jsr.2018-0078 31629325

[B20] Koo T. K. Li M. Y. (2016). A guideline of selecting and reporting intraclass correlation coefficients for reliability research. J. Chiropr. Med. 15 (2), 155–163. 10.1016/j.jcm.2016.02.012 27330520 PMC4913118

[B21] Li Y. Tsang R. C. Liu D. Ruan B. Yu Y. Gao Q. (2021). Applicability of cutoff scores of Chinese Cumberland Ankle Instability Tool and Foot and Ankle Ability Measure as inclusion criteria for study of chronic ankle instability in Chinese individuals. Phys. Ther. Sport 48, 116–120. 10.1016/j.ptsp.2020.12.021 33421739

[B22] Lin C. I. Khajooei M. Engel T. Nair A. Heikkila M. Kaplick H. (2021). The effect of chronic ankle instability on muscle activations in lower extremities. PLoS One 16 (2), e0247581. 10.1371/journal.pone.0247581 33617592 PMC7899370

[B23] McGee R. G. (2020). How to include patient-reported outcome measures in clinical trials. Curr. Osteoporos. Rep. 18 (5), 480–485. 10.1007/s11914-020-00611-5 32757118

[B24] Miklovic T. M. Donovan L. Protzuk O. A. Kang M. S. Feger M. A. (2018). Acute lateral ankle sprain to chronic ankle instability: A pathway of dysfunction. Phys. Sportsmed. 46 (1), 116–122. 10.1080/00913847.2018.1409604 29171312

[B25] Plisky P. J. Gorman P. P. Butler R. J. Kiesel K. B. Underwood F. B. Elkins B. (2009). The reliability of an instrumented device for measuring components of the star excursion balance test. N. Am. J. Sports Phys. Ther. 4 (2), 92–99. 21509114 PMC2953327

[B26] Portney L. G. (2020). Foundations of clinical research: Applications to evidence-based practice. F. A. Davis.

[B27] Rosen A. Ko J. Brown C. (2016). A multivariate assessment of clinical contributions to the severity of perceived dysfunction measured by the Cumberland ankle instability tool. Int. J. Sports Med. 37 (14), 1154–1158. 10.1055/s-0042-113464 27706549

[B28] Schober P. Boer C. Schwarte L. A. (2018). Correlation coefficients: Appropriate use and interpretation. Anesth. Analgesia 126 (5), 1763–1768. 10.1213/Ane.0000000000002864 29481436

[B29] Shao X. Wang Z. Luan L. Sheng Y. Yu R. Pranata A. (2022). Impaired ankle inversion proprioception during walking is associated with fear of falling in older adults. Front. Aging Neurosci. 14, 946509. 10.3389/fnagi.2022.946509 36247986 PMC9563849

[B30] Smith R. Rattanaprasert U. O'Dwyer N. (2001). Coordination of the ankle joint complex during walking. Hum. Mov. Sci. 20 (4-5), 447–460. 10.1016/s0167-9457(01)00062-8 11750672

[B31] Steffen T. Seney M. (2008). Test-retest reliability and minimal detectable change on balance and ambulation tests, the 36-item short-form health survey, and the unified Parkinson disease rating scale in people with parkinsonism. Phys. Ther. 88 (6), 733–746. 10.2522/ptj.20070214 18356292

[B32] Sturnieks D. L. St George R. Lord S. R. (2008). Balance disorders in the elderly. Neurophysiol. Clinique/Clinical Neurophysiol. 38 (6), 467–478. 10.1016/j.neucli.2008.09.001 19026966

[B33] Tao P. Shao X. Zhuang J. Wang Z. Dong Y. Shen X. (2021). Translation, cultural adaptation, and reliability and validity testing of a Chinese version of the freezing of gait questionnaire (FOGQ-CH). Front. Neurol. 12, 760398. 10.3389/fneur.2021.760398 34887830 PMC8649621

[B34] Vriend I. van Kampen B. Schmikli S. (2009). Ongevalsletsels en sportblessures in kaart gebracht. Ongevallen en Bewegen in Nederland 2006-2007.

[B35] Waterman B. R. Owens B. D. Davey S. Zacchilli M. A. Belmont P. J. Jr. (2010). The epidemiology of ankle sprains in the United States. J. Bone Jt. Surg. Am. 92 (13), 2279–2284. 10.2106/JBJS.I.01537 20926721

[B36] Wenning M. Gehring D. Lange T. Fuerst-Meroth D. Streicher P. Schmal H. (2021). Clinical evaluation of manual stress testing, stress ultrasound and 3D stress MRI in chronic mechanical ankle instability. BMC Musculoskelet. Disord. 22 (1), 198. 10.1186/s12891-021-03998-z 33596891 PMC7890850

[B37] Wilson B. R. Robertson K. E. Burnham J. M. Yonz M. C. Ireland M. L. Noehren B. (2018). The relationship between hip strength and the Y balance test. J. Sport Rehabil. 27 (5), 445–450. 10.1123/jsr.2016-0187 28714790

[B38] Xue X. a. Ma T. Li Q. Song Y. Hua Y. (2021). Chronic ankle instability is associated with proprioception deficits: A systematic review and meta-analysis. J. Sport Health Sci. 10 (2), 182–191. 10.1016/j.jshs.2020.09.014 33017672 PMC7987558

[B39] Yang N. Waddington G. Adams R. Han J. (2018). Translation, cultural adaption, and test-retest reliability of Chinese versions of the edinburgh handedness inventory and Waterloo footedness questionnaire. Laterality 23 (3), 255–273. 10.1080/1357650X.2017.1357728 28756732

[B40] Yu R. Yang Z. Witchalls J. Adams R. Waddington G. Han J. (2021). Kinesiology tape length and ankle inversion proprioception at step-down landing in individuals with chronic ankle instability. J. Sci. Med. Sport 24 (9), 894–899. 10.1016/j.jsams.2021.04.009 34016535

